# Diastolic Dysfunction and Severity of Cirrhosis in Nonalcoholic Cirrhotic Patients

**DOI:** 10.1155/2013/892876

**Published:** 2013-11-26

**Authors:** A. Salari, A. Shafaghi, M. Ofoghi, A. Saeidinia, F. Mansour-Ghanaei

**Affiliations:** ^1^Department of Cardiology, Guilan University of Medical Sciences (GUMS), Rasht, Iran; ^2^Division of Gastroenterology & Hepatology, Department of Gastroenterology, Gastrointestinal and Liver Diseases Research Center (GLDRC), Guilan University of Medical Sciences (GUMS), Razi Hospital, Sardar-Jangal Avenue, Rasht, Iran; ^3^Guilan University of Medical Sciences (GUMS), Rasht, Iran; ^4^Gastrointestinal and Liver Diseases Research Center (GLDRC), Guilan University of Medical Sciences (GUMS), Rasht, Iran

## Abstract

*Background*. In this study, we evaluated the association between diastolic dysfunction severity and severity of cirrhosis in nonalcoholic cirrhotic patients. *Methods*. This cross-sectional study was conducted on all nonalcoholic cirrhotic patients who were admitted in Rasht Razi hospital the Cancer of Guilan Province, north of Iran, from January 2011 to March 2012. Severity of cirrhosis was evaluated by Child-Pugh score. A 12-lead surface ECG and echocardiographic studies were performed. We used a HDI 3000 (Philips ATL, Bothell, WA, USA) equipped with 2 to 4 MHz probes. Diastolic function was determined by an expert cardiac sonographer. Data were analyzed by SPSS for win (version16). A *P* value less than 0.05 was considered significant. *Results*. Sixty-tree percent of patients were male. The mean age of patients was 52.78 ± 15.2 years. 22%, 38%, and 40% of patients were considered as child class A, B, and C, respectively. There was a significant relation between diastolic dysfunction and disease duration (*P* = 0.001), female gender (*P* = 0.004), age > 60 years (*P* = 0.045), and severity of cirrhosis (*P* = 0.048). On multivariate analysis, decreased E/A ratio (*P* = 0.03) and disease duration (*P* = 0.02) showed an independent significant relation. *Conclusion*. According to the relation between severity of cirrhosis and diastolic dysfunction, we recommend cardiac assessment in all child B and C cirrhotic patients.

## 1. Introduction 

Cirrhosis is a hepatic disease that presents in individuals aged 50–60 years, typically [1, 2]. Patients with liver cirrhosis are reported to have a hyperdynamic circulation, which is manifested as high cardiac output, decreased systemic vascular resistance, and widespread arterial vasodilatation, primarily [3, 4]. Based on many previous studies traditionally, cirrhosis is associated with cardiovascular abnormalities [5, 6].

Cirrhotic cardiomyopathy is the term used to describe a collection of characters expressive of abnormal heart structure and function in patients with cirrhosis [7, 8]. The term “cirrhotic cardiomyopathy” is generally defined by the following clinical criteria: (1) baseline increased cardiac output but blunted ventricular response to stimuli, (2) systolic and/or diastolic dysfunction, (3) absence of overt left ventricular failure at rest, and (4) electrophysiological abnormalities including prolonged QT interval on electrocardiography and chronotropic incompetence [9–11].

Many patients with cirrhosis exhibit various degrees of diastolic dysfunction. Diastolic relaxation is damaged in cirrhosis. Diastolic filling consists of two parts normally: rapid, early diastolic (active) relaxation and late diastolic (passive) filling. The first phase depends on the rate of ventricular relaxation, elastic ventricular recoil, the atrio-ventricular pressure gradient, and the passive elastic features of the left atrium and ventricle [12, 13].

The second phase formed on the basis of the strength of left atrial contraction and the stiffness of the left ventricle. Diastolic dysfunction occurs when the passive elastic traits of the myocardium are reduced to increased myocardial mass and changes in the extracellular collagen secondarily [14]. This leads to stiffening and hypertrophy of the left ventricle with decreased compliance and higher diastolic pressures at each diastolic volume. So, relatively small increases in intravascular volume can lead to elevations in diastolic pressures. Shifting this pressure into the left atrium and pulmonary venous system can lead to pulmonary edema [14–17].

Ventricular diastolic compliance and diastolic function can be assessed by measuring the velocity of blood flow from the left atrium to the left ventricle during early diastole (the E wave) and late diastole (the A wave) and calculating the E/A ratio by using the Doppler echocardiography [8]. In other words, determinants of a diastolic dysfunction on a Doppler echocardiogram are decreased E/A ratio the ratio of early to late (atrial) phases of ventricular filling and delayed early diastolic transmitral filling with prolonged deceleration and isovolumetric relaxation times [7, 18].

Many studies indicated that some level of diastolic dysfunction exists in most patients with cirrhosis [18–23]. Diastolic dysfunction may progress to systolic dysfunction, although this has not been directly shown in cirrhotic patients [13, 16, 23]. In this study, we evaluated the association of diastolic dysfunction severity and severity of cirrhosis in nonalcoholic patients who were admitted in Razi hospital, Rasht the Cancer of Guilan Province, north of Iran.

## 2. Material and Methods

This survey was conducted as a cross-sectional descriptive study. All nonalcoholic cirrhotic patients who were admitted from January 2011 to March 2012 were enrolled.

Severity of the cirrhosis was evaluated by Child-Pugh criteria and divided in three groups: A (mild), B (moderate), and C (severe).

This study project was approved by the ethics committee of the faculty of medicine, Guilan University of medical sciences, Iran. A written informed consent was obtained from each patient.

### 2.1. Electrocardiography

A 12-lead surface EGG was obtained from all subjects in the supine position immediately before echocardiography by using machine. The ECG was recorded at a paper speed of 50 mm/s. All measurements were made by one observer who was not aware of the patients' characteristics.

### 2.2. Echocardiography

Echocardiographic studies were performed using a HDI 3000 (Philips ATL, Bothell, WA, USA) equipped with 2 to 4 MHz probes allowing M-mode, two-dimensional, and pulsed Doppler measurements. An experienced cardiac sonographer performed the measurements. Echocardiography was performed according to the guidelines of American Society of Echocardiography [12].

The diastolic function was evaluated by the following indexes: left ventricular filling derived from mitral valve diastolic flow velocity curve, such as the ratio of E and A wave velocities (E/A); the atrial deceleration time (DT); and Early filling wave (Ea). The E wave corresponds to the peak initial mitral inflow velocity and the A wave to the velocity caused by atrial contraction. DT is associated with the left ventricular relaxation and corresponds to the slope of a straight line from the peak to half of the E velocity. The normal value is greater than or equal to 1.0 for the E : A ratio, less than or equal to 240 msec for ADT, and less than or equal to 110 msec for IRT. Diastolic dysfunction was present if one or two of the above parameters were present (Table 1).

### 2.3. Statistical Analysis

SPSS for Windows, version 16, was used for data analysis. The qualitative data were analyzed by chi-square and Fisher's exact test. Continuous variables are presented as mean ± standard deviation (SD); categorical variables are presented as percentages. We also use the multivariate analysis to reject confounders. *P* value <0.05 was considered significant.

## 3. Results 

Table 2 shows age, gender, and etiology of liver disease and child classification in our patients.  Table 3 shows the range and mean values of some laboratory results of study patients. The most common cause of cirrhosis in study population as mentioned in Table 2 was hepatitis C. The mean duration of diagnosis was 2.83 ± 4.02 years. There was a significant relation between diagnosis-duration and diastolic dysfunction (*P* = 0.001).

Forty nine percent of patients had normal diastolic function and 51% had diastolic dysfunction. There was a significant relation between gender and diastolic dysfunction (*P* = 0.004) and it was more frequent in female patients (Table 4).

There was a significant relation between diastolic dysfunction and age (*P* = 0.045). The age group of >60 years was the most frequent group of diastolic dysfunction. Up to 73.3% of cirrhotic patients in this group had some degree of diastolic dysfunction (Table 5).

The relation between severity of cirrhosis and diastolic dysfunction was significant in this study (*P* = 0.048) (Table 6). On multivariate analysis, decreased E/A ratio (*P* = 0.03) and disease duration (*P* = 0.02) showed an independent significant relation.

Table 7 shows the distribution of diastolic dysfunction severity between various causes of cirrhosis. Frequency of diastolic dysfunction in hepatitis C group is more frequent than the other. E/A and severity of cirrhosis had a significant correlation (*P* = 0.001).

## 4. Discussion 

Half of advanced cirrhotic patients had cardiac dysfunction. Diastolic dysfunction is a complex process that arises from numerous interrelated contributing factors such as pressure variations in the ventricle, cardiac preload and afterload, and ventricular relaxation and compliance [24]. The increased circulating blood volume, found in patient with cirrhosis, leads to a high cardiac preload and decreased peripheral vascular resistance with low cardiac afterload. The alteration in the diastolic function is likely due to an impaired ventricular relaxation. Diastolic dysfunction could be due to the stiffness of the ventricular wall as a result of cardiac hypertrophy described in cirrhotic cardiomyopathy [7, 25].

In this study, diastolic dysfunction in women was significantly more than men (*P* = 0.004). Similarly, in the study of Redfield et al. they showed that heart failure with normal ejection fraction in any ages is more frequent in women rather than men [26]. So, it could be concluded that the result may be due to the differences of sex hormones in each gender.

In our study, diastolic dysfunction was increased significantly by increasing duration of disease (*P* = 0.001). It was also seen in the study of Nasr et al. [27].

There was no correlation between means of E/A ratio in cirrhotic patients in comparison with control group and no evidence of ventricular hypertrophy. So, they showed that diastolic dysfunction could be due to ascites or liver dysfunction. In the presence of ascites, intrathoracic pressure increased and diaphragm shifted up. This could prevent enough relaxation of ventricle [27].

Correlation of age and diastolic dysfunction is important for managing and predicting the prognosis of disease. In our study, age group of >60 years had more diastolic dysfunction (*P* = 0.045). The same results can be seen in other studies like El-Adi et al. that concluded cardiac changes is age related in cirrhotic patients in comparison with control group and showed that incidence of diastolic dysfunction was increased by age [28]. Increasing age can affect cirrhosis and can exacerbate cardiac dysfunction.

Severity of cirrhosis and diastolic dysfunction had a significant correlation (*P* = 0.048). By increasing severity of cirrhosis from Child A to Child C, normal diastolic function was decreased and diastolic dysfunction was increased. Genovesi et al. showed that echocardiographic findings have no significant differences between groups [29]. In another study, by using Child-Pugh score and MELD score, moderate diastolic dysfunction and more severe cirrhosis are correlated significantly [27].

Achecar and Gonzalec-Tallon showed that diastolic dysfunction of left ventricle was exacerbated by liver disease [30]. The results of that study was similar to our study.

Prevalence of diastolic dysfunction in our study were 51% (44% mild, 6% moderate, and 1% severe). One study showed that 80%, 25%, and 24% of diastolic dysfunction were seen in patients who had severed, moderate, and mild liver fibrosis, respectively [27]. Another study showed that left ventricular diastolic dysfunction can be seen in 50% (25% grade I and 25% grade II) of cirrhotic patients [30]. In cirrhotic patients, patchy fibrosis may increase heart weight and also heart stiffness that affect ventricular filling and result in diastolic dysfunction [31]. The systemic circulation in patients with cirrhosis is hyperdynamic with an increased cardiac output and heart rate and a reduced systemic vascular resistance as the most pronounced alterations [31]. Expanding plasma volume increases cardiac preload and ultimately cardiac output in the presence of impaired cardiac contractility. Latent systolic and diastolic dysfunction with reduced work capacity is present and becomes in some patients manifested if the heart is challenged [32].

The presence of subclinical myocardial disease with cardiac dysfunction and decreased E/A ratio could be emphasized when patients' cardiac status will be improved by paracentesis of ascetic fluid [7]. On the other hand, after liver transplantation both physical activity and cardiac function seem to improve [33].

When the duration of cirrhosis was increased, liver cell failure was aggravated and cirrhosis becomes decompensated. In decompensated cirrhosis, there may be a further decrease in the arterial blood pressure owing to the unloading of baroreceptors and renal salt-water excretion is prolonged and incomplete [34]. On other hand, vascular compliance was abnormal in this setting [16]. Cardiac abnormalities such as heart stiffness due to fibrosis and circulatory problems may contribute to worsening of cardiac function in late stage of cirrhosis.

Cardiac dysfunction is a common complication of advanced cirrhosis that can make a variety of disturbances, specially QT interval prolongation and diastolic dysfunction. Diastolic dysfunction is correlated with gender and is more frequent in women. Duration of disease, increased age, and severity of cirrhosis can increase the severity of diastolic dysfunction.

Because of high prevalence of diastolic dysfunction in cirrhotic patients and risk of decompensation following invasive procedures, it could be suggested that all patients would be screened routinely by echocardiography before invasive procedures.

## 5. Conclusions

According to the relation between Child-Pugh score and diastolic dysfunction, we recommend cardiac assessment, especially echocardiographic evaluation in all Child B and Child C (decompensated) cirrhotic patients.

## Figures and Tables

**Table 1:** Diastolic dysfunction grades.

Grade 1: abnormal relaxation
E/A < 0.8
DT > 280 m·sec
E/Ea < 8
Grade 2: pseudonormalization
E/A = 1–1.5
DT = 160–240 m·sec
Ea < 7 cm/sec
Grades: 3 and 4
E/A > 2
E/Ea > 15
DT < 160 m·sec
Ea < 7 cm/sec

E/A: the velocity of the diastolic early filling wave (E) divided by the velocity of the late or atrial filling wave (A).

DT: deceleration time; Ea: early filling wave.

**Table 2:** Baseline data of study patients.

Characteristics		
Age (mean ± SD)		52.78 ± 15.2 (years)
Sex (M/F)		63/37

Cause	Cryptogenic	62 (62%)
	HBV	9 (9%)
	HCV	22 (22%)
	PSC	4 (4%)
	Immune	2 (2%)
	Budd-Chiari	1 (1%)

Child-Pugh classification	Child A	22 (22%)
	Child B	38 (38%)
	Child C	40 (40%)

**Table 3:** Mean and standard deviation of some laboratory results in study patients.

	Mean	Standard deviation
Creatinine (mg/dL)	1.03	0.66
Na (mmole/L)	139	5.24
K (mmole/L)	4.27	0.54
Ca (mmole/L)	8.17	0.65

**Table 4:** Distribution of diastolic function by gender.

Gender	Diastolic function						
	Normal function		Mild dysfunction		Moderate/sever dysfunction		Total
	Number	%	Number	%	Number	%	
Male	37	(58.7%)	25	(39.7%)	1	(1.6%)	63
Female	12	(32.4%)	19	(51.4%)	6	(16.2%)	37

Total	49	(49 %)	44	(44%)	7	(7%)	100

**Table 5:** Distribution of diastolic function in various age groups.

Age group	Diastolic function						
	Normal function		Mild dysfunction		Moderate/sever dysfunction		Total
	Number	%	Number	%	Number	%	
<40	15	(68.2%)	6	(27.3%)	1	(4.5%)	22
40–60	26	(54.2%)	19	(39.6%)	3	(6.2%)	48
>60	8	(26.7%)	19	(63.3%)	3	(10%)	30

Total	49	(49%)	44	(44%)	7	(7%)	100

**Table 6:** Distribution of diastolic function between child groups.

Child class	Diastolic function						
	Normal function		Mild dysfunction		Moderate/sever dysfunction		Total
	Number	%	Number	%	Number	%	
Child A	17	(77.3%)	4	(18.2%)	1	(4.5%)	22
Child B	17	(44.7%)	18	(47.4%)	3	(7.9%)	38
Child C	15	(37.5%)	22	(55%)	3	(7.5%)	40

Total	49	(49%)	44	(44%)	7	(7%)	100

**Table 7:** Distribution of diastolic function between various causes of cirrhosis.

Causes	Diastolic function						
	Normal function		Mild dysfunction		Moderate/sever dysfunction		Total
	Number	%	Number	%	Number	%	
Cryptogenic	27	(43.5%)	31	(50%)	4	(6.5%)	62
HBV	4	(44.4%)	5	(55.6%)	0	(0%)	9
HCV	13	(59.1%)	7	(31.8%)	2	(9.1%)	22
PSC	3	(75%)	1	(25%)	0	(0%)	4
Immune	1	(50%)	0	(0%)	1	(50%)	2
Budd-Chiari	1	(100%)	0	(0%)	0	(0%)	1

Total	49	(49%)	44	(44%)	7	(7%)	100
